# 4,5-Bis(1*H*-imidazol-1-ylmeth­yl)acridine monohydrate

**DOI:** 10.1107/S160053680903267X

**Published:** 2009-08-22

**Authors:** S. Thenmozhi, S. Ranjith, S. Raja, P. Rajakumar, A. SubbiahPandi

**Affiliations:** a Department of Physics, Presidency College (Autonomous), Chennai 600 005, India; b Department of Organic Chemistry, University of Madras, Chennai 600 025, India

## Abstract

In the title compound, C_21_H_17_N_5_·H_2_O, the dihedral angles between the acridine ring system and the imidazole rings are 78.8 (1) and 71.2 (1)°. The crystal packing is stabilized by O—H⋯N, C—H⋯O, C—H⋯π and π–π inter­actions [centroid–centroid separations = 3.732 (1) and 3.569 (1) Å].

## Related literature

For the biological activity of acridines, see: Talacki *et al.* (1974 ▶); Achenson (1956 ▶); Prasad Krishna *et al.* (1984 ▶); Asthana *et al.* (1991 ▶). For their anti­protozoal activity, see: Karolak-Wojciechowska *et al.* (1996 ▶). For the ability of acridine to inter­calate between the base-pairs of DNA, see: Neidle (1979 ▶); Fan *et al.* (1997 ▶). For acridine compounds in the treatment of Alzheimer’s disease, see: Bandoli *et al.* (1994 ▶). For their toxicity, see: Di Giorgio *et al.* (2005 ▶).
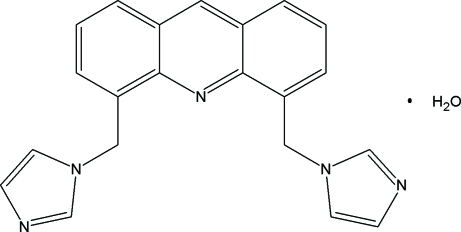


## Experimental

### 

#### Crystal data

C_21_H_17_N_5_·H_2_O
*M* *_r_* = 357.41
Monoclinic, 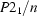

*a* = 14.3359 (6) Å
*b* = 6.9132 (3) Å
*c* = 17.7458 (8) Å
β = 92.895 (3)°
*V* = 1756.49 (13) Å^3^
*Z* = 4
Mo *K*α radiation
μ = 0.09 mm^−1^
*T* = 293 K
0.25 × 0.22 × 0.19 mm

#### Data collection

Bruker Kappa APEXII CCD diffractometer
Absorption correction: multi-scan (*SADABS*; Sheldrick, 1996 ▶) *T*_min_ = 0.978, *T*_max_ = 0.984
19520 measured reflections
4286 independent reflections
2652 reflections with *I* > 2σ(*I*)
*R*_int_ = 0.036

#### Refinement

*R*[*F* ^2^ > 2σ(*F* ^2^)] = 0.046
*wR*(*F* ^2^) = 0.145
*S* = 1.06
4286 reflections
252 parameters
H atoms treated by a mixture of independent and constrained refinement
Δρ_max_ = 0.19 e Å^−3^
Δρ_min_ = −0.20 e Å^−3^

### 

Data collection: *APEX2* (Bruker, 2004 ▶); cell refinement: *SAINT* (Bruker, 2004 ▶); data reduction: *SAINT*; program(s) used to solve structure: *SHELXS97* (Sheldrick, 2008 ▶); program(s) used to refine structure: *SHELXL97* (Sheldrick, 2008 ▶); molecular graphics: *ORTEP-3* (Farrugia, 1997 ▶); software used to prepare material for publication: *SHELXL97* and *PLATON* (Spek, 2009 ▶).

## Supplementary Material

Crystal structure: contains datablocks global, I. DOI: 10.1107/S160053680903267X/bt5021sup1.cif

Structure factors: contains datablocks I. DOI: 10.1107/S160053680903267X/bt5021Isup2.hkl

Additional supplementary materials:  crystallographic information; 3D view; checkCIF report

## Figures and Tables

**Table 1:** Hydrogen-bond geometry (Å, °)

*D*—H⋯*A*	*D*—H	H⋯*A*	*D*⋯*A*	*D*—H⋯*A*
C18—H18*B*⋯O1^i^	0.97	2.55	3.482 (3)	162
O1—H1*A*⋯N3	0.90 (3)	2.07 (3)	2.945 (3)	166 (3)
O1—H1*B*⋯N5^ii^	0.85 (3)	2.21 (3)	3.030 (3)	162 (3)
C7—H7⋯*Cg*1^iii^	0.93	2.69	3.577 (2)	159
C20—H20⋯*Cg*1^iv^	0.93	2.90	3.648 (2)	139

Symmetry codes: (i) 
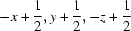
; (ii) 
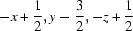
; (iii) 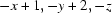; (iv) 
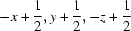
. *Cg*1 is the centroid of the N4/N5/C19–C21 ring.

**Fig. 1.**
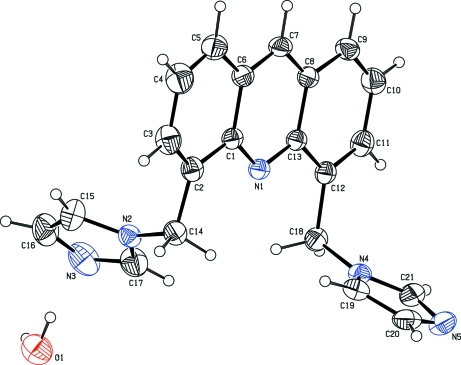
The structure of the title compound, showing the atom-numbering scheme and intramolecular hydrogen bond. Displacement ellipsoids are drawn at the 30% probability level.

**Fig. 2.**
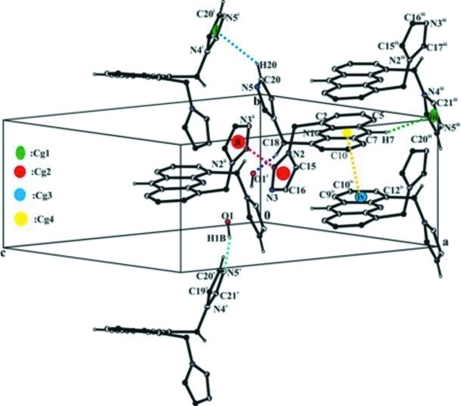
C–H···O, O–H···N, C–H···π and π—π interactions (dotted lines) in the title compound. Cg denotes ring centroid. [Symmetry code: (i) -1/2 - x, 1/2 + y, 1/2 - z; (ii) -x, 1 - y, -z; (iii) 1 - x, 2 - y, -z; (iv) 1 - x, 1 - y, -z; (v) 1/2 - x, 2 - y, -z]

## supplementary crystallographic information

### Comment

Acridines are found to have a wide range of biological activities, such as mutagenic, antitumour (Talacki *et al.*, 1974), antibacterial (Achenson, 1956), antiamoebic (Prasad Krishna *et al.*, 1984), hypersensitive, antiinflammatory and antiimplantation (Asthana *et al.*, 1991) activities. A drug containing the acridine moiety has been found to possess antiprotozoal activity (Karolak-Wojciechowska *et al.*, 1996). The ability of acridine to intercalate between the base-pairs of DNA is also well known (Neidle, 1979; Fan *et al.*, 1997). Acridine compounds are considered to be efficient drugs for the treatment of Alzheimer's disease (Bandoli *et al.*, 1994). Acridine derivatives have been shown to exert toxicity towards Plasmodium, Trypanosoma, and Leishmania parasites (Di Giorgio *et al.*, 2005). The imidazole group have found a wide range of applications in coordination chemistry as ligands, in medicinal chemistry and materials science. Against this background, and in order to obtain detailed information on molecular conformations in the solid state, an X-ray study of the title compound  has been carried out.

The two  imidazole groups are almost parallel to each other, the dihedral angle between the mean planes being 39.5 (1)°; these planes are inclined at 78.9 (1)° and 71.2 (9)° with respect to the mean plane of the acridine system. The pseudo-torsion angle N2–C14···.C18–N4 [-145.8 (2)°], resulting in both imidazole group being approximately bisected by the plane of the acridine system. The acridine ring system and imidazole rings are essentially planar, with maximum deviations of 0.062 (3), 0.006 (2) and 0.001 (2) Å, for atoms C4, N3 and C19, respectively.

The crystal packing is  stabilized by C–H···O, C–H···N, C–H···π (Table. 1) and π–π interactions with a *Cg*2···*Cg*2^ii^ and a *Cg*4···*Cg*3^iv^ separation of 3.732 (1)Å and 3.569 (1) Å, respectively (Fig.2; *Cg*2, *Cg*3 and *Cg*4 are the centroids of the N2/N3/C15–C17 imidazole ring, N1/C1/C6/C7/C8/C13 pyridine ring and C8–C13 benzene ring, respectively, symmetry code as in Fig. 2).

### Experimental

To a solution of imidazole, in the presence of acetonitrile (50 ml) and NaOH solution (7.5 ml) was added and stirred for 10 minutes, then 4,5-bis(bromomethyl) acridine in the presence of acetonitrile(20 ml) was added at once and stirred at room temperature for 48hrs. After completion of reaction, the solvent was evaporated in vaccum and the residue was extracted with CHCl_3_(300 ml). Single crystals suitable for the X-ray diffraction were obtained by slow evaporation of a solution of the title compound in methanol at room temperature.

### Refinement

H atoms of water were located is a difference fourier map, and were refined with distance restraints of O–H= 0.85 (3)Å. All other H atoms were fixed geometrically and allowed to ride on their parent atoms, with C–H = 0.93–0.98Å and with *U*_iso_(H) = 1.2*U*_eq_(C, N).

### Crystal data

**Table:**

C_21_H_17_N_5_·H_2_O	*F*(000) = 752
*M**_r_* = 357.41	*D*_x_ = 1.352 Mg m^−^^3^
Monoclinic, *P*2_1_/*n*	Mo *K*α radiation, λ = 0.71073 Å
Hall symbol: -P 2yn	Cell parameters from 4286 reflections
*a* = 14.3359 (6) Å	θ = 1.8–28.1°
*b* = 6.9132 (3) Å	µ = 0.09 mm^−^^1^
*c* = 17.7458 (8) Å	*T* = 293 K
β = 92.895 (3)°	Block, white crystalline
*V* = 1756.49 (13) Å^3^	0.25 × 0.22 × 0.19 mm
*Z* = 4	

### Data collection

**Table:**

Bruker Kappa APEXII CCD diffractometer	4286 independent reflections
Radiation source: fine-focus sealed tube	2652 reflections with *I* > 2σ(*I*)
graphite	*R*_int_ = 0.036
ω scans	θ_max_ = 28.1°, θ_min_ = 1.8°
Absorption correction: multi-scan (*SADABS*; Sheldrick, 1996)	*h* = −19→19
*T*_min_ = 0.978, *T*_max_ = 0.984	*k* = −9→8
19520 measured reflections	*l* = −23→23

### Refinement

**Table:**

Refinement on *F*^2^	Primary atom site location: structure-invariant direct methods
Least-squares matrix: full	Secondary atom site location: difference Fourier map
*R*[*F*^2^ > 2σ(*F*^2^)] = 0.046	Hydrogen site location: inferred from neighbouring sites
*wR*(*F*^2^) = 0.145	H atoms treated by a mixture of independent and constrained refinement
*S* = 1.06	*w* = 1/[σ^2^(*F*_o_^2^) + (0.0577*P*)^2^ + 0.3961*P*] where *P* = (*F*_o_^2^ + 2*F*_c_^2^)/3
4286 reflections	(Δ/σ)_max_ < 0.001
252 parameters	Δρ_max_ = 0.19 e Å^−^^3^
0 restraints	Δρ_min_ = −0.20 e Å^−^^3^

### Special details

**Table:**

Geometry. All e.s.d.'s (except the e.s.d. in the dihedral angle between two l.s. planes) are estimated using the full covariance matrix. The cell e.s.d.'s are taken into account individually in the estimation of e.s.d.'s in distances, angles and torsion angles; correlations between e.s.d.'s in cell parameters are only used when they are defined by crystal symmetry. An approximate (isotropic) treatment of cell e.s.d.'s is used for estimating e.s.d.'s involving l.s. planes.
Refinement. Refinement of *F*^2^ against ALL reflections. The weighted *R*-factor *wR* and goodness of fit *S* are based on *F*^2^, conventional *R*-factors *R* are based on *F*, with *F* set to zero for negative *F*^2^. The threshold expression of *F*^2^ > σ(*F*^2^) is used only for calculating *R*-factors(gt) *etc*. and is not relevant to the choice of reflections for refinement. *R*-factors based on *F*^2^ are statistically about twice as large as those based on *F*, and *R*- factors based on ALL data will be even larger.

### Fractional atomic coordinates and isotropic or equivalent isotropic displacement parameters (Å^2^)

**Table:**

	*x*	*y*	*z*	*U*_iso_*/*U*_eq_	
C1	0.31970 (12)	0.7854 (2)	−0.06027 (9)	0.0394 (4)	
C2	0.22370 (12)	0.8010 (3)	−0.08514 (10)	0.0463 (4)	
C3	0.20069 (15)	0.8387 (3)	−0.15855 (12)	0.0618 (5)	
H3	0.1382	0.8558	−0.1737	0.074*	
C4	0.26910 (17)	0.8528 (4)	−0.21250 (12)	0.0715 (6)	
H4	0.2513	0.8793	−0.2626	0.086*	
C5	0.35988 (16)	0.8283 (3)	−0.19224 (11)	0.0609 (5)	
H5	0.4041	0.8324	−0.2288	0.073*	
C6	0.38899 (13)	0.7960 (3)	−0.11539 (10)	0.0446 (4)	
C7	0.48111 (13)	0.7751 (3)	−0.09095 (10)	0.0466 (4)	
H7	0.5274	0.7779	−0.1258	0.056*	
C8	0.50554 (12)	0.7500 (2)	−0.01541 (10)	0.0414 (4)	
C9	0.59945 (12)	0.7289 (3)	0.01289 (12)	0.0509 (5)	
H9	0.6476	0.7313	−0.0203	0.061*	
C10	0.61933 (13)	0.7056 (3)	0.08691 (12)	0.0570 (5)	
H10	0.6811	0.6912	0.1048	0.068*	
C11	0.54661 (13)	0.7027 (3)	0.13806 (11)	0.0522 (5)	
H11	0.5617	0.6857	0.1892	0.063*	
C12	0.45576 (12)	0.7241 (2)	0.11476 (10)	0.0412 (4)	
C13	0.43189 (11)	0.7464 (2)	0.03633 (9)	0.0374 (4)	
C14	0.14924 (12)	0.7687 (3)	−0.03008 (11)	0.0490 (4)	
H14A	0.1671	0.8318	0.0173	0.059*	
H14B	0.0912	0.8261	−0.0496	0.059*	
C15	0.10166 (15)	0.4321 (3)	−0.06850 (13)	0.0663 (6)	
H15	0.0865	0.4554	−0.1193	0.080*	
C16	0.09479 (17)	0.2638 (3)	−0.03245 (16)	0.0750 (7)	
H16	0.0727	0.1496	−0.0545	0.090*	
C18	0.38042 (13)	0.7272 (3)	0.17082 (10)	0.0465 (4)	
H18A	0.3235	0.6737	0.1474	0.056*	
H18B	0.3992	0.6459	0.2135	0.056*	
C19	0.30224 (12)	1.0562 (3)	0.16560 (11)	0.0497 (5)	
H19	0.2643	1.0415	0.1219	0.060*	
C20	0.30963 (14)	1.2133 (3)	0.20991 (12)	0.0584 (5)	
H20	0.2765	1.3275	0.2013	0.070*	
C21	0.40165 (14)	1.0055 (3)	0.25943 (10)	0.0554 (5)	
H21	0.4454	0.9443	0.2917	0.067*	
N1	0.34184 (9)	0.76245 (19)	0.01343 (8)	0.0385 (3)	
N2	0.13497 (9)	0.5626 (2)	−0.01681 (8)	0.0446 (4)	
N3	0.12452 (15)	0.2830 (3)	0.04082 (14)	0.0815 (6)	
N4	0.36165 (9)	0.9226 (2)	0.19781 (8)	0.0427 (4)	
N5	0.37208 (13)	1.1834 (3)	0.26923 (10)	0.0658 (5)	
O1	0.04120 (12)	0.0382 (3)	0.15544 (11)	0.0766 (5)	
C17	0.14712 (15)	0.4659 (4)	0.04758 (12)	0.0668 (6)	
H17	0.1693	0.5221	0.0926	0.080*	
H1A	0.074 (2)	0.096 (5)	0.120 (2)	0.138 (14)*	
H1B	0.066 (2)	−0.070 (4)	0.1662 (16)	0.099 (11)*	

### Atomic displacement parameters (Å^2^)

**Table:**

	*U*^11^	*U*^22^	*U*^33^	*U*^12^	*U*^13^	*U*^23^
C1	0.0481 (9)	0.0282 (9)	0.0424 (9)	−0.0013 (7)	0.0082 (7)	0.0005 (7)
C2	0.0493 (10)	0.0365 (10)	0.0527 (11)	−0.0003 (7)	0.0009 (8)	0.0019 (8)
C3	0.0616 (12)	0.0634 (14)	0.0593 (12)	−0.0044 (10)	−0.0090 (10)	0.0072 (10)
C4	0.0830 (16)	0.0837 (18)	0.0469 (12)	−0.0154 (13)	−0.0069 (11)	0.0117 (11)
C5	0.0765 (14)	0.0628 (14)	0.0444 (11)	−0.0136 (11)	0.0120 (10)	0.0034 (9)
C6	0.0534 (10)	0.0353 (10)	0.0459 (10)	−0.0060 (7)	0.0119 (8)	−0.0001 (7)
C7	0.0533 (10)	0.0383 (10)	0.0501 (10)	−0.0047 (8)	0.0206 (8)	−0.0025 (8)
C8	0.0435 (9)	0.0277 (9)	0.0539 (10)	−0.0006 (7)	0.0115 (7)	−0.0023 (7)
C9	0.0434 (9)	0.0424 (11)	0.0681 (13)	0.0009 (8)	0.0150 (8)	−0.0049 (9)
C10	0.0434 (10)	0.0515 (13)	0.0756 (14)	0.0053 (8)	−0.0005 (9)	−0.0052 (10)
C11	0.0560 (11)	0.0450 (12)	0.0548 (11)	0.0033 (8)	−0.0039 (9)	−0.0028 (9)
C12	0.0468 (9)	0.0302 (9)	0.0469 (10)	0.0003 (7)	0.0044 (7)	−0.0014 (7)
C13	0.0419 (9)	0.0255 (8)	0.0456 (9)	0.0006 (6)	0.0082 (7)	−0.0013 (7)
C14	0.0443 (9)	0.0406 (11)	0.0623 (12)	0.0026 (8)	0.0027 (8)	−0.0001 (9)
C15	0.0788 (14)	0.0549 (14)	0.0635 (13)	−0.0027 (11)	−0.0115 (11)	−0.0049 (11)
C16	0.0743 (15)	0.0424 (13)	0.108 (2)	−0.0039 (10)	0.0035 (14)	−0.0016 (13)
C18	0.0569 (10)	0.0406 (11)	0.0423 (9)	−0.0052 (8)	0.0057 (8)	0.0042 (8)
C19	0.0440 (9)	0.0541 (12)	0.0519 (10)	0.0030 (8)	0.0122 (8)	0.0075 (9)
C20	0.0586 (12)	0.0549 (13)	0.0642 (13)	0.0068 (9)	0.0276 (10)	0.0016 (10)
C21	0.0568 (11)	0.0655 (14)	0.0445 (10)	−0.0020 (10)	0.0071 (8)	−0.0060 (9)
N1	0.0420 (7)	0.0303 (8)	0.0440 (8)	0.0003 (5)	0.0090 (6)	−0.0001 (6)
N2	0.0398 (7)	0.0426 (9)	0.0516 (9)	0.0010 (6)	0.0040 (6)	0.0018 (7)
N3	0.0780 (13)	0.0672 (15)	0.0993 (17)	−0.0087 (10)	0.0051 (12)	0.0298 (12)
N4	0.0448 (8)	0.0454 (9)	0.0388 (7)	−0.0028 (6)	0.0111 (6)	0.0010 (6)
N5	0.0769 (12)	0.0624 (13)	0.0598 (11)	−0.0014 (9)	0.0203 (9)	−0.0158 (9)
O1	0.0682 (10)	0.0828 (14)	0.0784 (12)	0.0017 (10)	0.0003 (9)	0.0099 (10)
C17	0.0725 (14)	0.0716 (17)	0.0562 (12)	−0.0135 (12)	0.0012 (10)	0.0141 (11)

### Geometric parameters (Å, °)

**Table:**

C1—N1	1.340 (2)	C14—N2	1.460 (2)
C1—C2	1.428 (2)	C14—H14A	0.9700
C1—C6	1.431 (2)	C14—H14B	0.9700
C2—C3	1.353 (3)	C15—C16	1.334 (3)
C2—C14	1.500 (2)	C15—N2	1.356 (3)
C3—C4	1.408 (3)	C15—H15	0.9300
C3—H3	0.9300	C16—N3	1.354 (3)
C4—C5	1.343 (3)	C16—H16	0.9300
C4—H4	0.9300	C18—N4	1.462 (2)
C5—C6	1.423 (3)	C18—H18A	0.9700
C5—H5	0.9300	C18—H18B	0.9700
C6—C7	1.377 (3)	C19—C20	1.342 (3)
C7—C8	1.379 (3)	C19—N4	1.362 (2)
C7—H7	0.9300	C19—H19	0.9300
C8—C9	1.421 (3)	C20—N5	1.363 (3)
C8—C13	1.434 (2)	C20—H20	0.9300
C9—C10	1.340 (3)	C21—N5	1.315 (3)
C9—H9	0.9300	C21—N4	1.338 (2)
C10—C11	1.416 (3)	C21—H21	0.9300
C10—H10	0.9300	N2—C17	1.327 (2)
C11—C12	1.355 (2)	N3—C17	1.310 (3)
C11—H11	0.9300	O1—H1A	0.91 (4)
C12—C13	1.425 (2)	O1—H1B	0.85 (3)
C12—C18	1.505 (2)	C17—H17	0.9300
C13—N1	1.338 (2)		
			
N1—C1—C2	119.20 (15)	N2—C14—H14A	109.4
N1—C1—C6	122.36 (15)	C2—C14—H14A	109.4
C2—C1—C6	118.44 (16)	N2—C14—H14B	109.4
C3—C2—C1	119.77 (17)	C2—C14—H14B	109.4
C3—C2—C14	120.60 (17)	H14A—C14—H14B	108.0
C1—C2—C14	119.60 (16)	C16—C15—N2	106.7 (2)
C2—C3—C4	121.58 (19)	C16—C15—H15	126.6
C2—C3—H3	119.2	N2—C15—H15	126.6
C4—C3—H3	119.2	C15—C16—N3	110.4 (2)
C5—C4—C3	120.52 (19)	C15—C16—H16	124.8
C5—C4—H4	119.7	N3—C16—H16	124.8
C3—C4—H4	119.7	N4—C18—C12	112.31 (14)
C4—C5—C6	120.63 (19)	N4—C18—H18A	109.1
C4—C5—H5	119.7	C12—C18—H18A	109.1
C6—C5—H5	119.7	N4—C18—H18B	109.1
C7—C6—C5	123.26 (17)	C12—C18—H18B	109.1
C7—C6—C1	117.86 (16)	H18A—C18—H18B	107.9
C5—C6—C1	118.88 (17)	C20—C19—N4	105.90 (18)
C6—C7—C8	120.78 (15)	C20—C19—H19	127.1
C6—C7—H7	119.6	N4—C19—H19	127.1
C8—C7—H7	119.6	C19—C20—N5	111.03 (19)
C7—C8—C9	123.10 (16)	C19—C20—H20	124.5
C7—C8—C13	117.74 (15)	N5—C20—H20	124.5
C9—C8—C13	119.16 (16)	N5—C21—N4	112.40 (19)
C10—C9—C8	120.65 (17)	N5—C21—H21	123.8
C10—C9—H9	119.7	N4—C21—H21	123.8
C8—C9—H9	119.7	C13—N1—C1	118.91 (14)
C9—C10—C11	120.20 (18)	C17—N2—C15	105.89 (18)
C9—C10—H10	119.9	C17—N2—C14	128.06 (17)
C11—C10—H10	119.9	C15—N2—C14	125.97 (17)
C12—C11—C10	122.02 (18)	C17—N3—C16	104.28 (19)
C12—C11—H11	119.0	C21—N4—C19	106.58 (17)
C10—C11—H11	119.0	C21—N4—C18	125.81 (16)
C11—C12—C13	119.33 (16)	C19—N4—C18	127.60 (15)
C11—C12—C18	120.72 (16)	C21—N5—C20	104.10 (17)
C13—C12—C18	119.95 (15)	H1A—O1—H1B	109 (3)
N1—C13—C12	119.07 (14)	N3—C17—N2	112.7 (2)
N1—C13—C8	122.30 (15)	N3—C17—H17	123.6
C12—C13—C8	118.62 (15)	N2—C17—H17	123.6
N2—C14—C2	111.13 (14)		
			
N1—C1—C2—C3	175.05 (17)	C9—C8—C13—N1	−178.93 (15)
C6—C1—C2—C3	−4.7 (3)	C7—C8—C13—C12	−178.90 (15)
N1—C1—C2—C14	−7.0 (2)	C9—C8—C13—C12	0.8 (2)
C6—C1—C2—C14	173.28 (16)	C3—C2—C14—N2	99.4 (2)
C1—C2—C3—C4	3.6 (3)	C1—C2—C14—N2	−78.6 (2)
C14—C2—C3—C4	−174.4 (2)	N2—C15—C16—N3	−1.1 (3)
C2—C3—C4—C5	0.2 (4)	C11—C12—C18—N4	89.8 (2)
C3—C4—C5—C6	−2.8 (4)	C13—C12—C18—N4	−89.38 (19)
C4—C5—C6—C7	−178.2 (2)	N4—C19—C20—N5	0.1 (2)
C4—C5—C6—C1	1.5 (3)	C12—C13—N1—C1	179.31 (14)
N1—C1—C6—C7	2.2 (2)	C8—C13—N1—C1	−1.0 (2)
C2—C1—C6—C7	−178.12 (16)	C2—C1—N1—C13	179.48 (15)
N1—C1—C6—C5	−177.52 (17)	C6—C1—N1—C13	−0.8 (2)
C2—C1—C6—C5	2.2 (2)	C16—C15—N2—C17	0.5 (2)
C5—C6—C7—C8	177.98 (18)	C16—C15—N2—C14	−176.70 (17)
C1—C6—C7—C8	−1.7 (3)	C2—C14—N2—C17	118.2 (2)
C6—C7—C8—C9	−179.63 (16)	C2—C14—N2—C15	−65.3 (2)
C6—C7—C8—C13	0.0 (2)	C15—C16—N3—C17	1.3 (3)
C7—C8—C9—C10	179.76 (18)	N5—C21—N4—C19	0.1 (2)
C13—C8—C9—C10	0.1 (3)	N5—C21—N4—C18	179.16 (15)
C8—C9—C10—C11	−0.3 (3)	C20—C19—N4—C21	−0.08 (19)
C9—C10—C11—C12	−0.4 (3)	C20—C19—N4—C18	−179.16 (15)
C10—C11—C12—C13	1.3 (3)	C12—C18—N4—C21	−92.4 (2)
C10—C11—C12—C18	−177.90 (18)	C12—C18—N4—C19	86.5 (2)
C11—C12—C13—N1	178.27 (15)	N4—C21—N5—C20	0.0 (2)
C18—C12—C13—N1	−2.6 (2)	C19—C20—N5—C21	0.0 (2)
C11—C12—C13—C8	−1.4 (2)	C16—N3—C17—N2	−1.0 (3)
C18—C12—C13—C8	177.73 (15)	C15—N2—C17—N3	0.4 (3)
C7—C8—C13—N1	1.4 (2)	C14—N2—C17—N3	177.43 (17)

### Hydrogen-bond geometry (Å, °)

**Table:**

*D*—H···*A*	*D*—H	H···*A*	*D*···*A*	*D*—H···*A*
C18—H18B···O1^i^	0.97	2.55	3.482 (3)	162
O1—H1A···N3	0.90 (3)	2.07 (3)	2.945 (3)	166 (3)
O1—H1B···N5^ii^	0.85 (3)	2.21 (3)	3.030 (3)	162 (3)
C7—H7···Cg1^iii^	0.93	2.69	3.577 (2)	159
C20—H20···Cg1^iv^	0.93	2.90	3.648 (2)	139

Symmetry codes: (i) −*x*+1/2, *y*+1/2, −*z*+1/2; (ii) −*x*+1/2, *y*−3/2, −*z*+1/2; (iii) −*x*+1, −*y*+2, −*z*; (iv) −*x*+1/2, *y*+1/2, −*z*+1/2.
